# A comparison of mdma-assisted psychotherapy to non-assisted psychotherapy in treatment-resistant PTSD: A systematic review and meta-analysis

**DOI:** 10.1192/j.eurpsy.2021.389

**Published:** 2021-08-13

**Authors:** B. Illingworth, D. Lewis, A. Lambarth, K. Stocking, J. Duffy, L. Jelen, J. Rucker

**Affiliations:** 1 Respiratory Medicine, Cambridge University Hospitals NHS Foundation Trust, Cambridge, United Kingdom; 2 Acute Medicine, Brighton & Sussex University Hospitals NHS Trust, Brighton, United Kingdom; 3 Clinical Pharmacology, University College London Hospitals NHS Foundation Trust, London, United Kingdom; 4 Centre For Biostatistics, University of Manchester, Manchester, United Kingdom; 5 Institute For Women’s Health, University College London, London, United Kingdom; 6 Centre For Affective Disorders, Institute of Psychiatry, Psychology & Neuroscience, King’s College London, London, United Kingdom

**Keywords:** MDMA, ptsd, Treatment-resistance, psychotherapy

## Abstract

**Introduction:**

Novel, evidence-based treatments are required for treatment-resistant post-traumatic stress disorder (PTSD). 3,4-Methylenedioxymethamphetamine (MDMA) has beneficially augmented psychotherapy in several small clinical trials.

**Objectives:**

To review the use of MDMA-assisted psychotherapy in treatment-resistant PTSD.

**Methods:**

Systematic searches of four databases were conducted from inception to February 2020. A meta-analysis was performed on trials which were double-blinded, randomised, and compared MDMA-assisted psychotherapy to psychotherapy and placebo. The primary outcomes were the differences in Clinician Administered PTSD Scale (CAPS-IV) score and Beck’s Depression Inventory (BDI). Secondary outcome measures included neurocognitive and physical adverse effects, at the time, and within seven days of intervention.

**Results:**

Four randomised controlled trials (RCTs) met inclusion criteria. When compared to active placebo, intervention groups taking 75mg (MD -46.90; 95% CI -58.78, -35.02), 125mg (MD -20.98; 95% CI -34.35, -7.61) but not 100mg (MD -12.90; 95% CI -36.09, 10.29) of MDMA with psychotherapy, had significant decreases in CAPS-IV scores, as did the inactive placebo arm (MD -33.20; 95% CI -40.53, -25.87). A significant decrease in BDI when compared to active placebo (MD -10.80; 95% CI -20.39, -1.21) was only observed at 75mg. Compared to placebo, participants reported significantly more episodes of low mood, nausea and jaw-clenching during sessions and lack of appetite after seven days.

**Conclusions:**

These results demonstrate potential therapeutic benefit with minimal physical and neurocognitive risk for the use of MDMA-assisted psychotherapy in TR-PTSD, despite little effect on Beck’s Depression Inventory. Better powered RCTs are required to investigate further.

**Disclosure:**

James Rucker has attended trial related meetings paid for by Compass Pathways Ltd.

